# Mesenchymal Stem Cells Transplantation in Intracerebral Hemorrhage: Application and Challenges

**DOI:** 10.3389/fncel.2021.653367

**Published:** 2021-03-24

**Authors:** Yu-hua Gong, Shi-lei Hao, Bo-chu Wang

**Affiliations:** Key Laboratory of Biorheological Science and Technology, Ministry of Education, College of Bioengineering, Chongqing University, Chongqing, China

**Keywords:** intracerebral hemorrhage, mesenchymal stem cells, different species, pathological microenvironment, optimizing strategy

## Abstract

Intracerebral hemorrhage (ICH) is one of the leading causes of death and long-term disability worldwide. Mesenchymal stem cell (MSC) therapies have demonstrated improved outcomes for treating ICH-induced neuronal defects, and the neural network reconstruction and neurological function recovery were enhanced in rodent ICH models through the mechanisms of neurogenesis, angiogenesis, anti-inflammation, and anti-apoptosis. However, many key issues associated with the survival, differentiation, and safety of grafted MSCs after ICH remain to be resolved, which hinder the clinical translation of MSC therapy. Herein, we reviewed an overview of the research status of MSC transplantation after ICH in different species including rodents, swine, monkey, and human, and the challenges for MSC-mediated ICH recovery from pathological microenvironment have been summarized. Furthermore, some efficient strategies for the outcome improvement of MSC transplantation were proposed.

## Introduction

Intracerebral hemorrhage (ICH) is one of the most devastating and life-threatening neurological diseases, which has led to high disability and mortality worldwide. Although ICH only accounts for ∼15% of all strokes, its mortality rate within 28 days is as high as ∼47%, and the surviving patients up to 25% are at risk of relapse ICH in the next 5 years (Chen Y. et al., 2020). Worse still, less than 12% of patients would have independent living ability at 1 year after ICH (van Asch et al., 2010). The incidence rate of ICH has not decreased with the development of medical and healthcare level, but shows an increasing trend in low- and middle-income countries (Wu and Anderson, 2020). This is probably due to the increased number of elderly people and the use of antiplatelet, anticoagulants, and thrombolytics (Carpenter et al., 2016; Wu et al., 2019). As of yet, considerable progress for ICH treatment has been made in preclinical research, however, there is still a lack of effective therapeutic strategies for the acute and long-term treatment of ICH in clinic, except for active rehabilitation.

Mesenchymal stem cell (MSC) therapy holds significant promise in regenerative medicine research and tissue engineering due to its unique properties, including extensive proliferation capacity, multiple differentiation potential, ease of isolation from various tissues, low immunogenicity, paracrine activity, immunomodulatory function, and fewer ethical disputes (Zheng et al., 2018). The application of MSC therapies has represented an exciting treatment for stroke, especially for ICH patients suffering from neurological deficits and motor dysfunction. To date, ICH researchers have conducted much pre-clinical and clinical research based on MSC treatment with promising results (Bedini et al., 2018; Gao et al., 2018).

However, the prognostic differences between individuals, the influence of pathological environment on the characteristics of implanted MSCs, and the long-term safety based on MSC therapies are undefined, which is still a non-negligible obstacle to clinical translation. When emphasizing that MSC replacement therapy is a very promising treatment option for ICH, the interaction between the pathological microenvironment of the individual and the implanted MSCs should also be strictly considered. Therefore, this review summarized the application of MSCs in the treatment of ICH in different species (e.g., rodent, swine, monkey, and human) and their mechanism of promoting neurological recovery. Meanwhile, we emphasized the existing challenges faced by MSCs planted into the microenvironment of ICH injured brain tissues, including the impacts of mass effect, iron overload, and oxidative stress. Furthermore, we further discussed the MSC-based potential therapeutic methods that might have an optimized clinical transformation effect in the treatment of ICH.

## Injury Mechanisms of ICH

The cascade brain injury initiated by ICH is conventionally described as two consecutive pathological processes. In the first stage of primary brain injury, the resulting hematoma from the ruptured vessel could form a persistent mass effect, and mechanically stretch and compress the brain tissue to cause brain tissue damage. Subsequently, the secondary brain injury was mainly caused by the hematoma and its degradation products (i.e., hemoglobin, heme, and iron), which could induce microglia activation, inflammatory response, oxidative stress, brain edema, and blood-brain barrier (BBB) leakage. The secondary brain injury will further lead to permanent death of brain tissue and severe neurological deficits (Qureshi et al., 2009; Wilkinson et al., 2018).

## Background of Mesenchymal Stem Cells

Mesenchymal stem cells represent a versatile class of multipotent stem cells that hold promise to directly regenerate damaged tissues due to their potential of differentiation into almost any end-stage lineage cells (Han et al., 2019). Bone marrow-derived MSCs were the firstly-discovered and extensively studied MSCs, although they also had been isolated and characterized from tissues including adipose, dermis, cord blood, peripheral blood, synovial fluid, umbilical cord, placenta, amniotic fluid, fetal tissues, dental pulp, periosteum, and skeletal muscle (Murphy et al., 2013; Bedini et al., 2018). Various minimally invasive procedures had been used to isolate and expand MSCs *in vitro*, including abdominoplasty, bone marrow aspiration, and placental collection (Sherman et al., 2019). Therefore, MSCs could be obtained in quantity from the patients or third-party donors. These characteristics of easy availability and no ethical concerns make them widely used in regenerative medicine research (Wang et al., 2018). It should be noted that although these cells have many similarities, MSCs still exist with different gene expression patterns, differentiation profile, and clinical application potential according to their origin (Ghanta et al., 2018).

## The Progress of MSC-Based Therapies in different Species After ICH

Although the exact mechanism behind MSC-based therapies was still largely ambiguous, numerous studies had been conducted in animals and humans, and some therapeutic effects had been achieved with various therapy mechanisms. However, differences about the focus point and outcomes of MSC treatment in ICH still exist among species. Detailed research findings of MSC-based ICH intervention in different species will be introduced in subsequent sections, and a summary of the relevant studies is reported in Table 1.

**TABLE 1:** The application of MSC-based therapy in different species of hemorrhage stroke.

Species (model)	Modeling approach	Amount	Treatment times (postbleed)	Route of treatment	Results	References
Rat (ICH)	Collagenase VII	2 × 10^6^ Cells	1, 3, 5 and 7 days	CA, CV, LV	Differentiated into neurons, astrocytes, and oligodendrocytes in the brain; The limb motor function and behavioral scores of the CA group and LV group were better than ICH-only and CV group	Zhang et al., 2006
Rat (ICH)	Collagenase IV	2 × 10^6^ Cells	3 days	IC	Enhanced the endogenous neurogenesis and differentiation; Reduced the mNSS scores	Otero et al., 2010
Rat (ICH)	Autologous blood	3, 5, or 8 × 10^6^ Cells	1 days	IV	Increased the immature neurons, neuronal migration, and synaptogenesis in the damaged brain region; Reduced tissue loss and NSS scores	Seyfried et al., 2006
Rat (ICH)	Collagenase VII	1 × 10^6^ Cells	1 h	IV	Increased proliferation, neural regeneration, anti-apoptotic molecules, G-CSF and BDNF expression; Reduced apoptosis, hemorrhage volume and mNSS scores	Wang S.P. et al., 2012
Rat (ICH)	Collagenase VII	5 × 10^5^ Cells	1 day	IC	Decreased apoptosis and inflammatory cell infiltration; Enhanced the endogenous neurogenesis, angiogenesis, BDFN and VEGF expression; Reduced the mNSS scores	Zhou et al., 2016
Rat (ICH)	Collagenase IV	5 × 10^6^ Cells	2 h	IV	Decreased apoptosis, brain water content, inflammatory cell infiltration, microglia activation, inflammatory-associated cytokines, BBB permeability, mNSS scores; Upregulated anti-inflammatory cytokines	Chen et al., 2015
Rat (ICH)	Collagenase VII	5 × 10^5^ Cells	2 days	IC	Reduced lesion volume, apoptosis, inflammatory factors Improved neurogenesis, angiogenesis, and behavioral recovery	Kim et al., 2015
Mice (ICH)	Collagenase A	1 × 10^6^ Cells	1 day	IV	Suppressed the acute inflammation; Improved neurological deficits; Reduced the mNSS scores	Kuramoto et al., 2019
Rat (SAH)	Endovascular perforation	3 × 10^6^ Cells	1 h	IV	Reduced brain water content, BBB disruption, neuronal injury, microglia activation, inflammatory cytokines expression Improved neurological function	Liu et al., 2019
Rat (ICH)	Collagenase VII	2 × 10^5^ Flk-1^+^ MSCs	1 day	IC	Reduced brain water content, hemorrhage volume, apoptosis, inflammatory cytokines, inflammatory cell infiltration, mNSS scores; Increased angiogenesis	Bao et al., 2013
Rat (ICH)	Collagenase VII	2 × 10^4^ Cells	1 day	IC	Increased angiogenesis, anti-inflammation Reduced apoptosis, injury volume, mNSS scores	Liao et al., 2009
Mice (ICH)	Collagenase VII	3 to 4 × 10^5^ Cells	2 days	IC	Reduced apoptosis, brain water content, AQP4 expression, inflammatory factors, mNSS scores	Zhang Y. et al., 2019
Rat (HICH)	Hemoglobin	1 × 10^5^ Cells	6 h	IC	Increased neuron content; Reduced BBB permeability, microglia activation, apoptosis, pro-inflammatory factors, the edema and mNSS scores.	Ding et al., 2017
Rat (IVH)	Maternal blood	1 × 10^5^ Cells	2 or 7 days	ICV	Decreased posthemorrhagic hydrocephalus, behavioral impairment, apoptosis, astrogliosis, inflammatory cytokines, and increased corpus callosum thickness, myelination at early stage of 2 days but not at 7 days	Park et al., 2016
Swine (TBI)	Cortical impact	4 × 10^13^ particles	9 h and 1, 5, 9, and 13 days	IV	Shortened neurologic recovery time; Reduced the mNSS scores	Williams et al., 2019
Monkey (ICH)	Autologous blood	1 to 5 × 10^6^ Cells	7 or 28 days	IC	Early treatment had more grafted cells uptake in the adjacent cortex and better results compared with later group MSCs treatment; Increased microvessel density and reduced neurologic deficit score	Feng et al., 2011
Human (ICH)	Spontaneous	20 × 10^6^ cells	More than 6 months	ICV	Improved neurological status; Observed no adverse events	Al Fauzi et al., 2016
Human (ICH)	Spontaneous	Mean of 4.57 × 10^7^ cells	1 year	IV	Improved neurological scores; Observed neither adverse event nor sign of de novo tumor development	Tsang et al., 2017
Human (SAH)	Aneurysmal	10 × 10^7^ cells	3 days	IV	The patient achieved a rapid and favorable recovery	Brunet et al., 2019
Human (IVH)	Premature	5 or 10 × 10^6^ cells	7 days	ICV	Decreased pro-inflammatory cytokines, VEGF in CSF; Observed no dose-limiting toxicities, serious adverse effects or mortality	Ahn et al., 2018
Human (ICH)	Spontaneous	1 × 10^6^ cells	NA	CS	Decreased the brain edema, serum nerve injury marker molecules level; Increased the BDNF, NGF, and endothelial progenitor cells level	Zhang, 2016
Human (ICH)	Spontaneous	1.8 × 10^8^ cells	14 days	IC	MSCs therapy had better functional outcomes in 5 years fellow-up Hu-MSCs graft had better outcomes than autologous BMSCs graft	Chang et al., 2016

*ICH, intracerebral hemorrhage; SAH, subarachnoid hemorrhage; HICH, hypertensive intracerebral hemorrhage; IVH, intraventricular hemorrhage; TBI, traumatic brain injury; CSF, cerebrospinal fluid; CA, carotid artery; CV, cervical vein; LV, lateral ventricle; IC, intracerebral; IV, intravenous; ICV, intracerebroventricular; CS, cavum subarachnoidale; NSS, Neurological Severity Score; mNSS, modified NSS; NA, not available; G-CSF, granulocyte colony-stimulating factor; BDNF, brain-derived neurotrophic factor; VEGF, vascular endothelial growth factor; NGF, nerve growth factor; BBB, blood-brain barrier; AQP4, aquaporin-4; Hu-MSCs, human umbilical cord-derived mesenchymal stem cells; BMSCs, bone-marrow derived mesenchymal stem cells.*

### MSC Transplantation in Rodents

At present, plenty of experimental research has been conducted on the treatment of rodent ICH with MSC transplantation, probably due to the versatility of rodent models. Large amounts of evidence confirmed that MSC transplantation is a promising treatment for neuronal recovery after ICH (Ma et al., 2015; Hu et al., 2016; Zheng et al., 2018). In view of the neurological function improvement effect of MSCs in ischemic rat brain, it was no doubt that the first MSC application in the rat ICH model also showed significant improvement in motor function (Zhang et al., 2006). To investigate the role of MSCs in ICH therapy, BrdU-labeled MSCs were delivered into the brain through carotid artery (CA), cervical vein (CV), or lateral ventricle (LV), respectively, at days 1, 3, 5, and 7 following ICH. Thereafter, BrdU-labeled MSCs were found in the brain of CA and LV delivery groups except CV injection. The majority of labeled MSCs could migrate into the ipsilateral cortex, bleeding area, and hippocampus. Additionally, double staining revealed that MSCs mainly differentiated into neurons in the hippocampus, while MSCs differentiated into neurons and astrocytes around the bleeding area. In the ipsilateral cortex, MSCs could differentiate into neurons, astrocytes, and oligodendrocytes. Taken together, these results demonstrated that the functional improvement of MSCs in ICH was probably related to their ability to differentiate into appropriate cell types. Furthermore, the nestin and doublecortin positive cells had been observed in the subventricular zone (SVZ) and nearby the lesion zone in the ICH animals received MSCs, indicating that the MSC transplantation could also enhance endogenous neurogenesis and differentiation (Otero et al., 2010). Therefore, MSC therapy could mediate the transplanted and endogenous neurogenesis to reorganize the lost neuronal circuity after ICH. The observation of immature neurons and decreased tissue loss of striatum further confirmed the organizational restructuring role of MSCs in ICH (Seyfried et al., 2006, 2008).

The molecular mechanisms underlying MSC-mediated neurogenesis behavior was partly attributed to the trophic properties of MSCs, which could secrete neurotrophic and other chemokines to promote transplanted or endogenous stem cells proliferation and differentiation (Murphy et al., 2013; Baker et al., 2019). Previous studies demonstrated that tail vein delivered bone marrow-derived MSCs had increased the expression of brain-derived neurotrophic factor (BDNF), which was a crucial growth factor for the growth and differentiation of central nervous systems (Wang S.P. et al., 2012). Knockout of BDNF in MSCs had significant suppression in the effective treatment results of transplanted cells in intraventricular hemorrhage (IVH) (Ahn et al., 2017). Vascular endothelial growth factor (VEGF), which was closely related to angiogenesis, was also significantly upregulated in ICH brain tissues with transplanted MSCs (Zhou et al., 2016). In addition, glial cell-derived neurotrophic factor (GDNF) was also recognized as an important neurotrophic factor in the growth, differentiation, development, maintenance, and injury repair for several types of neurons. Not only transfection but also drug-induced increase of GDNF could significantly enhance the functional improvement of grafted MSCs on ICH (Yang et al., 2011; Lee et al., 2015; Deng et al., 2019). Furthermore, delivered hypoxia-preconditioned MSCs could reduce the tissue loss of the ipsilateral striatum, and enhance neuroregeneration and neurological functional recovery after ICH, which was attributed to the neuronal nourish and protect effect of MSC-mediated upregulation of GDNF, VEGF, and BDNF (Sun et al., 2015).

Neuronal cells insult caused by the primary brain injury and secondary brain injury after ICH was mainly located around the hematoma. Therefore, whether endogenous stem cells or exogenous stem cells could migrate to the perihematoma was a key factor for neuronal remodeling. To promote cells viability and migration, platelet-rich plasma (PRP)-derived scaffold was employed to deliver MSCs, and the contained fibronectin, fibrin, and adhesive proteins in the scaffold were expected to augment cell interaction and promote cell migration. Apparently, (PRP)-derived scaffold increased the biologic activity of donor cells and its integration in the injured tissue (Vaquero et al., 2013).

Although the potential neural recovery mechanism of MSCs in ICH is still largely undefined, apparently, it is not limited to the differentiation and remodeling of brain tissue. In fact, mechanisms involved in tissue repair, such as neurogenesis, angiogenesis, anti-inflammation, and anti-apoptosis, were all suggested to be associated with the functional recovery effect of transplanted MSCs in ICH (Chen et al., 2015; Kim et al., 2015; Xie et al., 2016; Kuramoto et al., 2019; Liu et al., 2019). It was well characterized that ICH-induced inflammation response was a key factor in secondary brain injury, which involved microglia activation, and infiltrating neutrophils release could induce BBB breakdown, vasogenic edema, and apoptosis of glia and neurons (Keep et al., 2012). Thus, a previous study had proposed a hypothesis that the behavioral recovery improved by MSC therapy might derive from the inhibition of inflammation cascade in ICH. In order to verify this hypothesis, Flk-1^+^ MSCs was introduced for ICH (Bao et al., 2013). Results showed that Flk-1^+^ MSCs-treatment dramatically decreased the neurons apoptosis, brain water content, inflammatory cells expression (e.g., microglia and neutrophils), and the mRNA levels of inflammatory mediators (e.g., IL-1β, IL-2, IL-4, IL-6, and TNF-α). Moreover, umbilical cord-derived MSCs administration had statistically increased vessel density, and reduced microglial activation and leukocytes infiltration at 3 days after ICH compared to the control group. These suggested that, except for neuronal regeneration, the underlying mechanisms of MSCs to accelerate neurological function recovery after ICH were also attributed to its effects of promoting angiogenesis and inhibiting inflammation (Liao et al., 2009). Other therapeutic mechanisms revealed that MSC transplantation-mediated inflammation suppression and down-regulation of aquaporin-4 (AQP4) protein expression could alleviate brain edema of ICH (Zhang Y. et al., 2019). The mechanism of transplanted MSCs inhibiting inflammation might be partly involved in the inhibition of iNOS expression (Ding et al., 2017). In addition, the microglial M2 polarization mediated by the secretory factors, insulin-like growth factor-1 (IGF-1) of stem cells, was considered to be a possible mechanism for stem cells improved hemorrhage stroke prognosis (Chen et al., 2019; Sun et al., 2020). Taken together, these studies demonstrated that anti-inflammation is a crucial mechanism involved in MSCs-mediated functional and tissue recovery after ICH.

Moreover, Adipose-derived stem cells administration rats of ICH found significantly decreased brain water content, brain atrophy, and apoptosis, indicating the multiple mechanisms of MSCs therapy in ICH recovery (Kim et al., 2007). Furthermore, other studies had demonstrated that MSCs transplantation improved BBB integrity after ICH, which was associated with the increased expression of TNF-α stimulated gene/protein 6 (TSG-6) and tight junction proteins (claudin-5 and zonula occludens-1) (Chen et al., 2015; Choi et al., 2018). As hypertension is the most common cause of cerebral hemorrhage clinically, a spontaneously hypertensive model was created to evaluate the long-term neuroprotective effects of MSCs in ICH, which would have great value for pre-clinical study. This study demonstrated that MSC treatment could reverse the BBB permeability and improve the neurological function after hypertensive ICH (Wang et al., 2015). For stem cell therapy, the conventional mode of administration is injection of cells into the brain directly or through blood vessels. However, the method of cells being injected into the brain is invasive, and regarding the method of vascular administration, it has been demonstrated that there were only a few cells homing to the brain or had embolization and possible tumorigenesis risk (Li P. et al., 2019; Mello et al., 2020). Thus, a novel way of intranasal route was applied for MSC transplantation therapy in collagenase IV injected mice (Sun et al., 2015). In addition, MSC-derived exosomes were also considered as a promising alternative strategy to solve the obstacle in MSC application (Bedini et al., 2018).

### MSC Transplantation in Swine

Although different from the etiology of spontaneous ICH, the rehabilitation research of traumatic cerebral hemorrhage also had an important reference value for the clinical transformation of ICH intervention, especially in large animals. Exosomes were membrane-enclosed nanovesicles that contained numerous molecular constituents including cellular proteins, lipids, microRNAs and mRNAs, and had the properties of low immunogenicity, BBB penetrability, intercellular communication and neural regeneration mediation (Yang et al., 2017). Meanwhile, due to the neuroprotective effect of MSCs-generated exosomes in small animal traumatic brain injury (TBI) model, they were also highly expected in the treatment of traumatic cerebral hemorrhage in large animals (Zhang Y. et al., 2015). When Yorkshire swine were treated with MSCs-derived exosomes, they appeared to have faster neurologic recovery and obvious reduction of neurologic injury (Williams et al., 2019). The benefit outcomes in large animal models would provide a reliable evidence for the clinical application of preclinical research.

### MSC Transplantation in Monkeys

The first study of MSC treatment for primates was conducted in an ICH model of intracranial autologous injected *macaca fascicularis monkey* (Feng et al., 2011). The MSC-treated group showed significantly improved neurologic deficit and microvessel density compared with the control group. Additionally, the serial ^18^F-FDG PET scans found that the ^18^F-FDG uptake in the adjacent cortex of the early treatment was increased significantly when compared with control group during recovery phase, and better treatment results were obtained in the MSC early treatment group (7 days) compared with late treatment group (28 days). However, the optimal timing of MSC transplantation for severe IVH in newborn rat pups was considered to be at early stage of 2 days instead of late at 7 days after induction (Park et al., 2016). Therefore, when extrapolating preclinical data into clinical trials, the narrow therapeutic time window of MSC transplantation for ICH intervention should be determined. Meanwhile, it is also worth noting that stereotaxical delivery of MSCs to the outside of the right putamen of *macaca fascicularis monkey* could induce the local tissue inflammation and necrosis, although this damage might repair over time (Feng et al., 2014). Nevertheless, a total of four intravenous injections of MSCs (1 × 10^7^ cells/kg) with 2 weeks interval did not affect the general health of monkeys (Wang Y. et al., 2012). These studies suggested that the delivery modes and therapeutic time window of MSCs applied for ICH were key factors associated to prognosis and required further elucidation.

### MSC Transplantation in Humans

Mesenchymal stem cells have been extensively studied and confirmed as an effective treatment strategy for animal cerebral hemorrhage models, however, less research has been investigated in human cerebral hemorrhage. The first application of MSCs in humans was a case report about two persistent vegetative state patients of hemorrhagic stroke, and then they were followed-up with for a 1 year period (Al Fauzi et al., 2016). Patients were intraventricularly injected with MSCs (20 × 10^6^ cells/2.5 mL) three times at intervals of 1 month using an Ommaya reservoir. The National Institute of Health Stroke Scale (NIHSS) scores of the two patients indicated that the functional status was obviously improved. A placebo-controlled study also found that two deliveries of MSCs (2 × 10^6^ cells/3 mL) 1 month apart could significantly improve the neuro-restoration and clinical prognosis of ICH patients with severe disability (Tsang et al., 2017). In another case report of an 80-year-old SAH patient with a past medical history (i.e., diabetes, hypertension, and cardiac bypass) found that MSC transplantation gradually improved the consciousness of the patient at 3 weeks after SAH, and restored the ability of speaking and self-care (Brunet et al., 2019). Consistently, this procedure might provide a safe, feasible and effective treatment for patients with clinical cerebral hemorrhage in the future, and enhance the confidence in the treatment of patients with cerebral hemorrhage.

Based on previous experimental and clinical benefit, MSC transplantation was also carried out on premature infants to explore its safety and efficacy for severe IVH (Ahn et al., 2018). A total of nine premature infants were enrolled in this clinical trial, and different doses of MSCs were injected into the lateral ventricle (5 × 10^6^ cells/kg; 1 × 10^7^ cells/kg) on average at twelve postnatal days. All patients could tolerate the procedure well, and there were no immediate complications (i.e., allergies or death) which occurred within 6 h after transplantation. In these infant patients, four showed continual regression and improvement, and five received shunt placement due to the progressive ventriculomegaly before discharge. In most MSC transplanted patients, the inflammatory factors such as IL-6, TGF-β1/2, TNF-α, and IL-1β all exhibited a decreasing trend compared to before transplantation, that was consistent with that in animal experiments (Bao et al., 2013). Different from preclinical research, VEGF showed a reduced expression after MSC therapy, which was probably related to the up-regulation of baseline VEGF expression caused by hemorrhage insult (Tang et al., 2007). Meanwhile, Safety evaluation results showed both doses of MSCs did not induce the serious adverse events and dose-limiting toxicity, and the mortality was zero even in grade 4 IVH, which implied this strategy was safe when the dose was controlled within 1 × 10^7^ cells/kg of MSCs. As severe IVH and subsequent posthemorrhagic hydrocephalus could cause long-term neurological disorders in surviving preterm infants, children with neurological sequelae underwent a noteworthy clinical trial in which they were treated with bone marrow-derived mononuclear cells (BMMNCs) that might have the synergic effect of hematopoietic stem cells (HSCs) and MSCs (Liem et al., 2019). Except for no observed any adverse side effect, BMMNC transplantation had made great progress in many aspects of neurodevelopment, such as motor function, fine motor skill, personal social, motor adaptive, and language skills, demonstrating the BMMNCs treatment effectiveness in promoting the social interaction and self-care ability of these children. This improvement in human-specific physiological functions was difficult to evaluate in preclinical animal studies, and the functional ameliorate illustrated that the transplanted stem cells had availably differentiated into region specific cell types and integrated into neural network appropriately.

Because of the mechanical damage of hematoma to brain tissue, clot evacuation was a commonly used intervention in the clinical treatment of ICH. Therefore, the combined therapy of MSC transplantation followed by minimally invasive hematoma removal was supposed as a promising treatment for ICH. Compared with surgical treatment, the combined group significantly decreased the brain edema, and increased the BDNF, NGF, and Endothelial progenitor cells level (Zhang, 2016). In a case report of a female patient with a past medical history of hypertension, the combined treatment also observed improved neurologic function consistent with preclinical studies (Zhang Q. et al., 2015; Zahra et al., 2020). However, there were discrepancies among combined therapy with respect to prognosis in different sources of transplanted MSCs, which might be due to differences in the viability, proliferation potential, and neurogenic efficacy of the tested cell line. A retrospective analysis of up to 5-years follow up had documented that the patients who received human umbilical cord-derived MSCs in the combined therapy group showed better outcome on functional recovery and complications than the bone-marrow MSCs combined therapy group and control group (Chang et al., 2016). Overall, the MSCs transplantation had promise to be safely and effectively applied in clinical ICH treatment, even so, the underlying regulatory mechanism, the optimal implantation time, and larger clinical trials still need to be further considered in future research.

## Differences Between Preclinical and Clinical Studies

Mesenchymal stem cell transplantation had demonstrated the positive therapeutic effects of facilitating tissue repair and prognosis of ICH in animal research and clinical trials. However, significant research differences still existed in the process of transforming preclinical research to clinical application. The uncertainty contained in the differences of this research might affect the therapeutic mechanisms of transplanted MSCs and the stability of clinical benefits from many aspects. Whether the therapeutic mechanisms of MSC therapy reported in animal studies could effectively translate to the complex pathological environment of ICH patients was also unknown.

### Research Subjects

Actually, the differences between experimental research and clinical trials had already appeared in the screening of research subjects. Healthy rodents were the most versatile research group in preclinical studies. However, patients with ICH usually had a variety of other diseases, because ICH often occurred in the antiplatelet, anticoagulants, and thrombolytics users or elder peoples (Brunet et al., 2019). In addition, the reported ICH patients also included a strict inclusion and exclusion criteria, which did not exist in the healthy animal research subjects (Ahn et al., 2018). Moreover, the small sample size and case report in clinical studies further increased the publication bias. Most importantly, none of the current animal ICH models could reproduce the actual pathological process of ICH. Taken together, in the process of extrapolating the trial results to clinical application, it was necessary to be further verified from the research objects, model establishment, sample size, and other aspects.

### MSC Preparation

It is well known that the cell source, extraction, and expansion process of MSCs are direct factors associated with the biological characteristics and differentiation fate of implanted cells. In the rodent studies, MSCs derived from different sources (bone marrow, adipose, umbilical cord, placenta, dental pulp, and cord blood) had been used for ICH treatment and displayed an improved prognosis (Choi et al., 2018; Chen et al., 2019; Liu et al., 2019; Zhang Y. et al., 2019; Chen K.H. et al., 2020). Nevertheless, this trend of cell source balance had been tilted in human research. Except for a small amount of MSCs derived from umbilical cord or cord blood (Chang et al., 2016; Ahn et al., 2018), most of the transplanted MSCs were extracted through autologous bone marrow puncture in the clinical studies (Liem et al., 2019; Zahra et al., 2020). Different from this, the bone marrow-MSCs in the rodent studies were mainly derived from allogeneic femur and tibia. In addition, the MSCs transplantation in patients were all homogeneous, and MSCs implanted in rodents were also derived from heterogeneous (Chen K.H. et al., 2020). However, no research had focused on the influence of MSCs source or extraction differences on ICH treatment. Considering the risk and time consumption of autologous MSCs extraction, the immunogenicity of allogeneic MSCs, and the high cost of maintaining cells viability, optimized and practical MSCs product preparation still needed to be clarified in research to ensure the stability and predictability during transformation for clinical treatment.

### Registration and Preliminary Results

Unlike animal research, registration was introduced to comprehensively and objectively record all clinical trials which had been conducted. However, many clinical studies did not report the registration information, and only a few studies had documented the registration number of ClinicalTrials.gov (Ahn et al., 2018; Zahra et al., 2020). Although the improved prognosis of MSC treatment had been widely reported, the results of animal studies were mainly histopathological examination and neurological evaluation (Liu et al., 2019), and the clinical studies were more about blood composition testing and neurological function scores (Zhang, 2016; Brunet et al., 2019). In fact, clinical results about MSCs treated ICH were more of a case report for the patients (Liem et al., 2019). The lack of control studies not only made the results occasional, but also greatly compromised the quality of clinical trials. Therefore, any conclusive reports about clinical therapeutic mechanisms or effects might be too early and not rigorous. The clinical application of MSCs for ICH treatment still needed more controlled and statistically significant clinical trials.

## Challenges of ICH Microenvironment on MSCs Therapy

**FIGURE 1:**
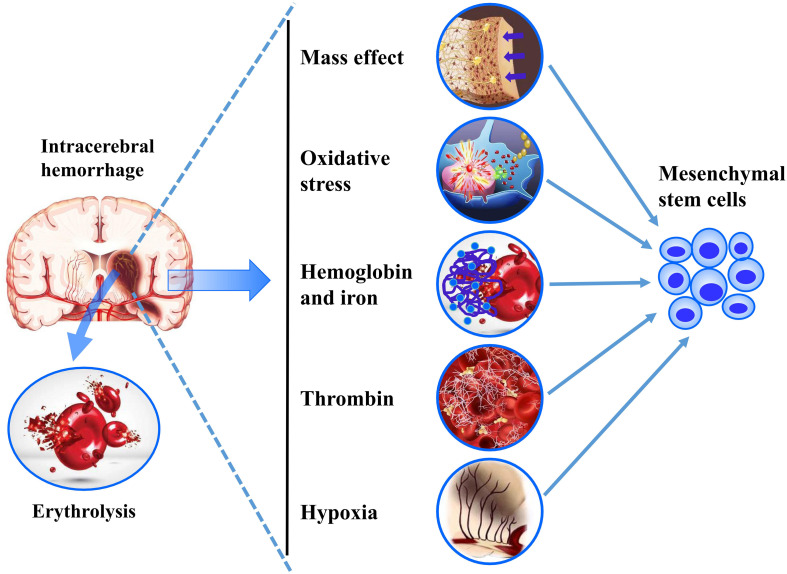
A summarization of the challenges of ICH microenvironments on MSC transplantation.

The extracellular environment plays an important role in the proliferation, differentiation, and clinical prognosis of transplanted MSCs. In fact, the biomimetic microenvironment not only helps to maintain the advantages of MSCs (e.g., proliferation, differentiation, and immunoregulatory properties), but also helps to retain their phenotype, metabolism, adhesion, and response signal to surrounding factors (Kim H. et al., 2019). The central nervous system refers to a complicated system containing neurons, glial cells, blood vessels, and extracellular matrix, which closely interacts with the transplanted MSCs and determines its fate (Dooves et al., 2016). However, the composition of the brain tissue microenvironment with hemorrhage infiltration becomes more complex and changeable, including mass effect, erythrocyte lysate, thrombin, free radicals, cytotoxic, excitotoxic, inflammatory effects, and nitric oxide (Babu et al., 2012; Gao et al., 2018). Meanwhile, many different stimulations had been recognized as the initiators of the stress-induced premature senescence of MSCs, including mechanical stress, osmotic stress, ionizing radiation, reactive oxygen species, and hypoxia (Zhou et al., 2020). Therefore, the extracellular microenvironment of perihematoma should be strictly evaluated when exploring effective and translatable MSCs therapies for hemorrhage stroke (Figure 1).

### Mass Effect

Mass effect is the main primary brain injury mechanism in ICH, which refers to the mechanical stretching and compression of the surrounding brain tissue by the hematoma (Wilkinson et al., 2018). Moreover, in the limited space of the skull, hematoma can lead to increased intracranial pressure and edema-derived mass effect (Guo et al., 2017). These would all result in a dynamic change and increase of mechanical stress in the intracranial microenvironment. So, the transplanted MSCs would sit in a microenvironment of mechanobiology with multiple complex mechanisms. Our studies had suggested that mechanical pressure enhanced the stretch-activated ion channel Piezo-2 expression *in vitro* and *vivo* as early as 8 h after ICH (Guo et al., 2018; Gong et al., 2019). *In vitro* data from adult *Drosophila* midgut feeding with indigestible food to generate mechanical loaded showed an increase enteroendocrine cells generation through Piezo protein upregulation, these suggested that the existence of stem cells in the fly midgut could, through mechanical signals, sense to modulate proliferation and differentiation (He et al., 2018). Mechanobiology, as the inductive niche of stem cells, could regulate their developmental processes and self-renewal (Vining and Mooney, 2017).

### Thrombin

Thrombin, derived from blood after ICH, is an essential component of the coagulation cascade, which has been observed to induce the brain injury through activating the protease-activated receptors (PAR) (Gao et al., 2014; Mao et al., 2016). Although thrombin is a proven tissue damage mechanism in ICH, thrombin-activated platelet-rich plasma was reported to provide a higher proliferation rate and MSC marker expression in long-term cultured MSCs (Kocaoemer et al., 2007). Thrombin-preconditioned MSCs displayed a five-fold acceleration of MSC-derived extracellular vesicles biogenesis and a two-fold enrichment of their cargo contents, these were also regulated via PAR-mediated intracellular signaling pathways (i.e., ERK1/2, AKT, Rab5, and EEA-1) (Sung et al., 2019). Meanwhile, thrombin also could promote fibronectin secretion of MSCs via PAR-mediated ERK1/2 activation (Chen et al., 2014), and the fibronectin-formed adsorption force could regulate the transmission of the cell traction force and the lineage specifications of MSCs (Lin et al., 2018). Additionally, in the newborn rat model of unilateral carotid artery ligation, thrombin preconditioned human Whartong’s jelly-derived MSC transplantation significantly enhanced the anti-inflammatory, anti-astroglial, anti-apoptotic effects, and neurological recovery (Kim Y.E. et al., 2019). Apparently, thrombin might have multiple undetermined influence mechanisms on the characteristics of transplanted MSCs during hemorrhage stroke.

### Iron Overload

Intracerebral hemorrhage, except for the primary damage of mass effect, intraparenchymal blood, and its degradation products (hemoglobin, heme, and iron), could continue to insult the brain tissue through inducing the cytotoxic, excitotoxic, oxidative, and inflammatory effects (Babu et al., 2012). Among these, iron overload was considered as an essential factor to increase the lipid peroxidation, lethal reactive oxygen species (ROS) production and neuronal ferroptosis (Li et al., 2017; Wan et al., 2019). Worse still, the iron overload microenvironment also posed a fatal threat to the therapy of stem cells transplantation. It had been demonstrated that ferric ammonium citrate treatment was capable of markedly reducing the proliferation and pluripotency, and inducing apoptosis and senescence in MSCs (Yang et al., 2016). In addition, iron accumulation was found to elevate the ROS level and apoptosis of MSCs, suggesting that the damage mechanism of iron overload in MSCs transplantation may be related to oxidative stress (Lu et al., 2013; Yuan et al., 2019). Notably, interventions to reduce mitochondrial ROS accumulation could significantly reduce the apoptosis of MSCs (Yang et al., 2016; Yu et al., 2018). Besides, the *in vitro* and *in vivo* iron overload were found to have the ability to inhibit the osteogenic commitment and differentiation of MSCs in a dose-dependent manner (Balogh et al., 2016). Therefore, these results indicated that the secondary iron overload microenvironment after ICH would affect not only the apoptosis but also the differentiation of transplanted MSCs.

### Oxidative Stress

Although ROS is an inevitable product of the defense system and normal cellular metabolism, the imbalanced homeostasis of the oxidation-reduction system can significantly create disruption to the blood-brain barrier, cell death and structural damage. And that ROS aggregation could be induced by the metabolic products of hematoma, inflammatory cells, and excitatory amino acids after ICH (Qu et al., 2016). Meanwhile, ROS had been extensively considered as an important factor of senescence in MSCs (Ko et al., 2012; Zhou et al., 2020). The oxidative stress formed by sub-lethal doses of hydrogen peroxide could considerably reduce proliferation rate, and accelerate telomere attrition, and induce senescence-associated β-galactosidase expression and senescent morphological features (Brandl et al., 2011). The nuclear factor erythroid-2 related factor 2 (Nrf2) is a critical molecule to protect MSCs against oxidative stresses. It had been reported that the MSCs with transient expression of Nrf2 had the ability to resist the hypoxic and oxidative stress induced cell death and apoptosis (Mohammadzadeh et al., 2012). Oxidative stress preconditioning could protect the MSCs vitality through activating the Nrf2 pathway and upregulating its downstream target of the superoxide dismutase, catalase, and HO-1 (Zhang F. et al., 2019). Moreover, many strategies had been explored to avoid cell apoptosis and senescence initiated by oxidative stress, including pioglitazone, vaspin, ganoderic acid D, and vitamin E (Bhatti et al., 2018; Hu et al., 2019; Zhu et al., 2019; Xu et al., 2020). Taken together, before MSCs could be reliably and effectively used as hemorrhage stroke therapy, it is necessary to better understand the multiple microenvironmental regulatory mechanisms driving transplanted MSCs to form repair effect.

## Potential Strategies for MSCs Treatment Enhancement After ICH

The above description highlights the critical function of ICH pathological microenvironment in determining the MSCs fate. The knowledge regarding mass effect and oxidative stress induced MSCs senescence provides a potential mechanism for the failure of MSCs-mediated clinical benefit in ICH. This indicated that effective therapeutic effect of MSCs on ICH required to strict regulation of the impacts of microenvironment on grafted MSCs. Facing the challenges of applying MSCs in the treatment of ICH, several optimizing strategies had been established and showed potential feasibility.

### Preconditioning Treatments

Studies had shown that preconditioning of MSCs could effectively resist the impact of transplantation microenvironment and increase the regenerative potential of cells, although the underlying mechanism was unknown. Hypoxic preconditioning, pharmacological agents, and trophic factors exposure were commonly used to improve culture expansion conditions, enhance survival and proliferation, and avoid the possible changes of MSCs potency (Hu and Li, 2018; Nahhas and Hess, 2018). In the ICH mice model, the transplantation of hypoxic preconditioned MSCs or neural stem cell significantly increased the grafted-cells survival and the behavioral performance (Sun et al., 2015; Wakai et al., 2016). Additionally, apocynin preconditioned MSCs obviously improved the hematoma expansion, neuronal death, brain edema, and therapeutic efficacy in the acute stage of bacterial collagenase induced rat ICH model compared with native MSCs (Min et al., 2018).

Other than pharmacological and molecular preconditioning of the MSCs, the resistance of MSCs to the microenvironment could also be achieved using miRNA. MiRNA based treatments had the characteristics of delayed MSC senescence and multidimensional targets. Given the significant function of miRNA-126 in promoting angiogenesis, the miRNA-126 transfected MSCs were injected into the collagenase-induced ICH rats, leading to decreased brain water content and improved neurological score (Wang et al., 2020). In addition, the CX3CR1 overexpressed MSCs increased the viability and migration ability of transplanted MSCs, and improved the sensory and motor functions of the collagenase induced mice ICH model (Li G. et al., 2019). Moreover, GDNF-transfected MSCs could improve the neurological function of experimental ICH rats through enhancing neurotrophic factor secretion (Deng et al., 2019). Therefore, miRNA-based MSC therapy was expected to purposefully regulate the proliferation and differentiation ability of MSCs in the pathological microenvironment of ICH.

### Optimizing ICH Microenvironment

Creating a standardized and locally beneficial microenvironment is another strategy to maintain the repair potential of transplanted MSCs. Iron overloaded and oxidative stress damage after ICH were recognized as not only critical damage factors for ICH, but also important causes of MSC senescence and apoptosis (Wan et al., 2019; Yuan et al., 2019). To improve the iron overload microenvironment after ICH, an injectable core-shell hydrogel was fabricated for ICH in situ therapy. The outer shell hydrogel with quick degradation property was loaded with iron chelator to eliminate iron overload, and the inner core hydrogel loaded with MSCs and growth factors displayed an improved MSCs survival and differentiation (Gong et al., 2020). In addition, surgical evacuation was a commonly used clinical approach to remove hematoma, and the benefits of combined treatment with MSCs transplantation might associate with the improvement of the transplanted cells microenvironment (Zhang Q. et al., 2015; Chang et al., 2016).

### Exosomes Therapies

The focus of MSC therapy is cell replacement and neural reorganization, however, its embolization and possible tumorigenesis limitations make exosomes a potential alternative. Significantly, exosomes are even suggested to be the main therapeutic mechanism of MSCs treatment (Han et al., 2018). Exosomes are nano-sized extracellular vesicles, which can be isolated from the supernatant of cultured cells in exosome-free medium through centrifugation and other methods (Bedini et al., 2018). Recently, treatment strategies based on exosomes of MSCs have been widely reported in many diseases. The potential mechanism of exosomes treatment in the ICH mainly comprises the anti-apoptotic, neurogenesis, angiogenesis, and anti-inflammation effects through its miRNAs (Cai et al., 2020; Duan et al., 2020).

The autologous blood ICH rats showed that the apoptotic and degenerative neurons in the miR-133b-modified MSC-derived exosomes treatment group was significantly reduced. It was believed that the neuroprotection effect of exosomes on ICH was mainly reflected in the anti-apoptotic effect of miR-133b through mediating RhoA and ERK1/2/CREB signaling pathway (Shen et al., 2018). The MSC-derived miR-206-knockdown exosomes were also confirmed to improve brain edema and neurological deficit in early brain injury of SAH rats, which was probably related to the suppression of neuronal apoptosis via BDNF/TrkB/CREB signaling (Zhao et al., 2019). Except for anti-apoptotic, the effects of neurogenesis and angiogenesis were also observed in the MSCs-derived exosomes treated ICH rats. The exosomes therapy remarkably increased the newly generated endothelial cells around the hematoma, mature neurons in the SVZ, and myelin in the striatum (Han et al., 2018). Moreover, the MSC-derived exosomes therapy was found to significantly alleviate early brain injury of SAH through restraining the HMGB1-TLR4 pathway activation and generating the anti-apoptosis and anti-inflammation effects (Xiong et al., 2020). Collectively, treatments based on exosomes are expected to provide a broader therapeutic strategy for ICH intervention.

## Conclusion

Mesenchymal stem cell-based ICH treatments provide a unique opportunity to improve ICH outcome through multimodal therapeutic action. Multi-species preclinical studies have demonstrated promising evidence that MSCs can promote the neural network reconstruction and neurological function recovery through neurogenesis, angiogenesis, anti-inflammation, and anti-apoptosis mechanisms. Meanwhile, human clinical trials have further confirmed the safety and efficacy of MSCs-based therapy after ICH, and the potential to improve patient prognosis. However, additional studies aimed to better understand the pathological microenvironment related to the successful implantation of MSCs after ICH (e.g., mass effect, thrombin, oxidative stress, and hematoma degradation products) will ultimately augment the therapeutic effect and enhance the reliability and stability of clinical transformation. Furthermore, MSC-derived exosomes treatment might represent a promising way to deal with the complex pathological microenvironment of hemorrhagic stroke.

## Author Contributions

YG searched the literature and wrote the manuscript. BW and SH critically revised the manuscript. All authors have made a substantial, direct and intellectual contribution to the work, and approved it for publication.

## Conflict of Interest

The authors declare that the research was conducted in the absence of any commercial or financial relationships that could be construed as a potential conflict of interest.
